# SARS-CoV-2 variants are associated with different clinical courses in children with MIS-C

**DOI:** 10.1007/s12519-023-00778-8

**Published:** 2023-12-22

**Authors:** Andres F. Moreno Rojas, Emelia Bainto, Helen Harvey, Adriana H. Tremoulet, Jane C. Burns, Kirsten B. Dummer

**Affiliations:** 1 Department of Pediatrics, Division of Pediatric Cardiology, University of California San Diego, and Rady Children’s Hospital, 3020 Children’s Way MC 5004, San Diego, CA 92123 USA; 2 Kawasaki Disease Research Center, La Jolla, CA USA; 3 Department of Pediatrics, Division of Pediatric Critical Care, University of California San Diego, and Rady Children’s Hospital, San Diego, CA USA; 4 Department of Pediatrics, University of California San Diego, and Rady Children’s Hospital, San Diego, CA USA

**Keywords:** Complications, COVID-19, MIS-C, SARS-CoV-2 variants

## Abstract

**Background:**

Recent infection with SARS‑CoV‑2 in children has been associated with multisystem inflammatory syndrome in children (MIS-C). SARS‑CoV‑2 has undergone different mutations. Few publications exist about specific variants and their correlation with the severity of MIS-C.

**Methods:**

This was a single-center, retrospective study including all patients admitted with MIS-C at Rady Children’s Hospital-San Diego between May 2020 and March 2022. Local epidemiologic data, including viral genomic information, were obtained from public records. Demographics, clinical presentation, laboratory values, and outcomes were obtained from electronic medical records.

**Results:**

The analysis included 104 pediatric patients. Four MIS-C waves were identified. Circulating variants in San Diego during the first wave included clades 20A to C. During the second wave, there were variants from clades 20A to C, 20G, 21C (Epsilon), 20I (Alpha), and 20J (Gamma). The third wave had Delta strains (clades 21A, 21I, and 21J), and the fourth had Omicron variants (clades 21K, 21L, and 22C). MIS-C presented with similar symptoms and laboratory findings across all waves. More patients were admitted to the pediatric intensive care unit (PICU) (74%) and required inotropic support (63%) during the second wave. None of the patients required mechanical circulatory support, and only two required invasive ventilatory support. There was no mortality.

**Conclusions:**

The various strains of SARS-CoV-2 triggered MIS-C with differing severities, with the second wave having a more severe clinical course. Whether the differences in disease severity across variants were due to changes in the virus or other factors remains unknown.

## Introduction

In April 2020, a new syndrome characterized by severe inflammation, multiorgan dysfunction, and features resembling Kawasaki disease and toxic shock syndrome in previously healthy children was described. These patients shared a recent infection with SARS-CoV-2. This condition is now recognized as multisystem inflammatory syndrome in children (MIS-C) [1, 2].

Throughout the pandemic, the coronavirus has undergone mutations that have changed its infectivity, transmission, and severity of the clinical presentation. Studies have reported that acute infection with the Omicron variant had less severe clinical outcomes, while Delta caused more severe disease [3, 4]. When looking at various time periods and linking the cases to the prevalent circulating variants, some studies suggested an effect on the presentation and severity of MIS-C, while others have not shown differences [5–14]. However, no reports have described specific local circulating variants and their correlation with MIS-C severity. We evaluated how the SARS-CoV-2 variants affected the clinical presentation and severity of MIS-C in San Diego County.

## Methods

This retrospective study was conducted at Rady Children’s Hospital, San Diego, a single tertiary care pediatric center that serves a population base of approximately 3.5 million residents. The Institutional Review Board at the University of California, San Diego, approved prospective data collection, and parents and participants gave signed informed consent or assent as appropriate.

All patients diagnosed with MIS-C between April 2020 and March 2022 were included in the evaluation. MIS-C was diagnosed using the 2020 CDC MIS-C case definition, as all the patients were diagnosed prior to the updated CDC definition for MIS-C (November 2022) [15]. The MIS-C/Kawasaki disease (KD) team developed a clinical pathway to standardize the approach and management of the patients. Two physicians (AHT and JCB) confirmed the history and clinical presentation, adjudicated each diagnosis and were primarily responsible for treatment in collaboration with pediatric intensivists when necessary. Our center participated in the MIS-C Comparative Effectiveness Study (MISTIC) trial (NCT04898231) that started on December 22, 2020. The trial randomized the patients who received IVIG but clinically warranted further anti-inflammatory therapy to one of three treatment arms (infliximab, steroids, or anakinra) and allowed for rerandomization to one of the two remaining arms if clinically warranted [16].

Four MIS-C waves were identified during the study period. We defined the “first wave” from April 2020 to August 2020, the “second wave” from September 2020 to April 2021, the “third wave” from May 2021 to December 2021, and the “fourth wave” from January 2022 to March 2022. The separation of the waves was based on analyzing the variation in the number of MIS-C cases at our institution, with at least 4 weeks with no cases between the waves.

COVID-19 case data for San Diego County were obtained from https://searchcovid.info/dashboards/epidemiology/ and https://outbreak.info [17]. The CDC established national surveillance for SARS-CoV-2 genomic sequencing in November 2020 [18]. The first reports appeared in December 2020, and previous genomic information in the US about circulating variants is limited. The SARS‑CoV‑2 genomic data of the sequences isolated in San Diego were obtained from The Scripps Research Institute and included samples obtained before December 2020 [19, 20]. The genomic data were analyzed for variant classification (World Health Organization (WHO) and Phylogenetic Assignment of Named Global Outbreak (PANGO) lineages) and clade assignment (Nextstrain clade) using Nextclade CLI v2.9.1. Since the WHO classification includes mostly variants of interest and concern and the PANGO lineage system is extensive and detailed, Nextstrain clades were used to facilitate the analysis of the circulating SARS‑CoV‑2 variants during the waves. Detailed information about Nextstrain clades can be found at https://clades.nextstrain.org/ [21]. Monthly frequency was analyzed, and variants with a frequency below 2% were excluded from the graphics. The results and discussion included only variants with a frequency above 5%.

Demographic characteristics (age, sex, race, weight, height), comorbidities (obesity, hypertension, asthma, diabetes mellitus type 1 and type 2, congenital heart disease, chronic kidney disease, autoimmunity), presenting clinical signs (fever, rash, conjunctival injection, erythema of the lips, oral mucosa or pharynx, cervical lymphadenopathy, erythema of the hands, abdominal pain, emesis, and diarrhea), laboratory results at the time of admission and worst value during hospitalization [white blood cell count, hemoglobin, platelet count, erythrocyte sedimentation rate, C reactive protein, ALT, AST, GGT, sodium, albumin, creatinine, ferritin, D dimer, fibrinogen, brain natriuretic peptide (BNP), and troponin I], volume of intravenous fluid boluses (with normal saline, ringer’s lactate, plasmalyte, 3% hypertonic saline or 5% albumin) administered in the first 24 hours, use of intravenous immunoglobulin (IVIG), time from initial evaluation to IVIG administration, additional anti-inflammatory therapies (steroids, anakinra, infliximab), need for PICU admission, length of stay in the PICU, use of inotropic and respiratory support, and echocardiogram results were obtained from the electronic medical records. The IVIG protocol was a single 2 g/kg dose over 8–12 hours. Additional anti-inflammatory therapies were used per the MISTIC trial.

Because the severe cases of MIS-C present in shock, the main criteria for admission to the PICU were hemodynamic instability, cardiac dysfunction requiring a continuous infusion of vasoactive medications, or respiratory insufficiency requiring more than a high-flow nasal cannula. Overweight/obese was defined as BMI > 85 percentile for age and gender for patients over two years of age and weight for length more than two standard deviations above the median for patients under two years of age. Severe obesity was defined as BMI > 99 percentile for age and gender. CDC growth charts were used. Laboratory results were analyzed as continuous variables and converted into categorical values with the following definitions: leukopenia: WBC < 5 × 10^9^/L. Lymphopenia: lymphocyte count < 4.5 × 10^9^/L if aged < 8 months or < 1.5 × 10^9^/L if aged ≥ 8 months. Thrombocytopenia: Platelet count < 150 × 10^9^/L. Hypoalbuminemia: Albumin < 3.5 g/dL. Hyponatremia: Sodium < 135 mmol/L. Elevated ferritin: ≥ 300 ng/mL. Elevated NLR: Neutrophil to lymphocyte ratio > 3.5. Elevated troponin I > 0.05 ng/mL. Elevated BNP ≥ 1000 pg/mL. For the analysis, cardiac dysfunction was defined as a left ventricular ejection fraction (LVEF) < 55% on echocardiogram.

The primary analysis was focused on finding differences between the different waves. Data analysis was performed in R (https://www.r-project.org/). Normal distribution was not assumed; the Mann‒Whitney *U* test was used to compare continuous variables among two groups, while the Kruskal‒Wallis test was used when comparing more than two groups. Pearson’s Chi-square or Fisher’s exact test was performed to compare nominal variables. If a test was significant, a post hoc analysis with Bonferroni correction was performed.

## Results

From April 2020 to March 2022, 109 patients were diagnosed with MIS-C. Five patients were excluded because the final diagnosis did not meet the CDC criteria for MIS-C upon adjudication. All cases occurred in patients who had never received a vaccine against SARS-CoV-2. All patients had serologic evidence of previous infection with SARS-CoV-2, mostly by a positive IgG nucleocapsid antibody. Only one case was negative for the nucleocapsid but positive for the anti-spike protein antibody. All patients underwent PCR testing for SARS-CoV-2 on admission, but only 12/104 (12%) had a positive result.

**Fig. 1:**
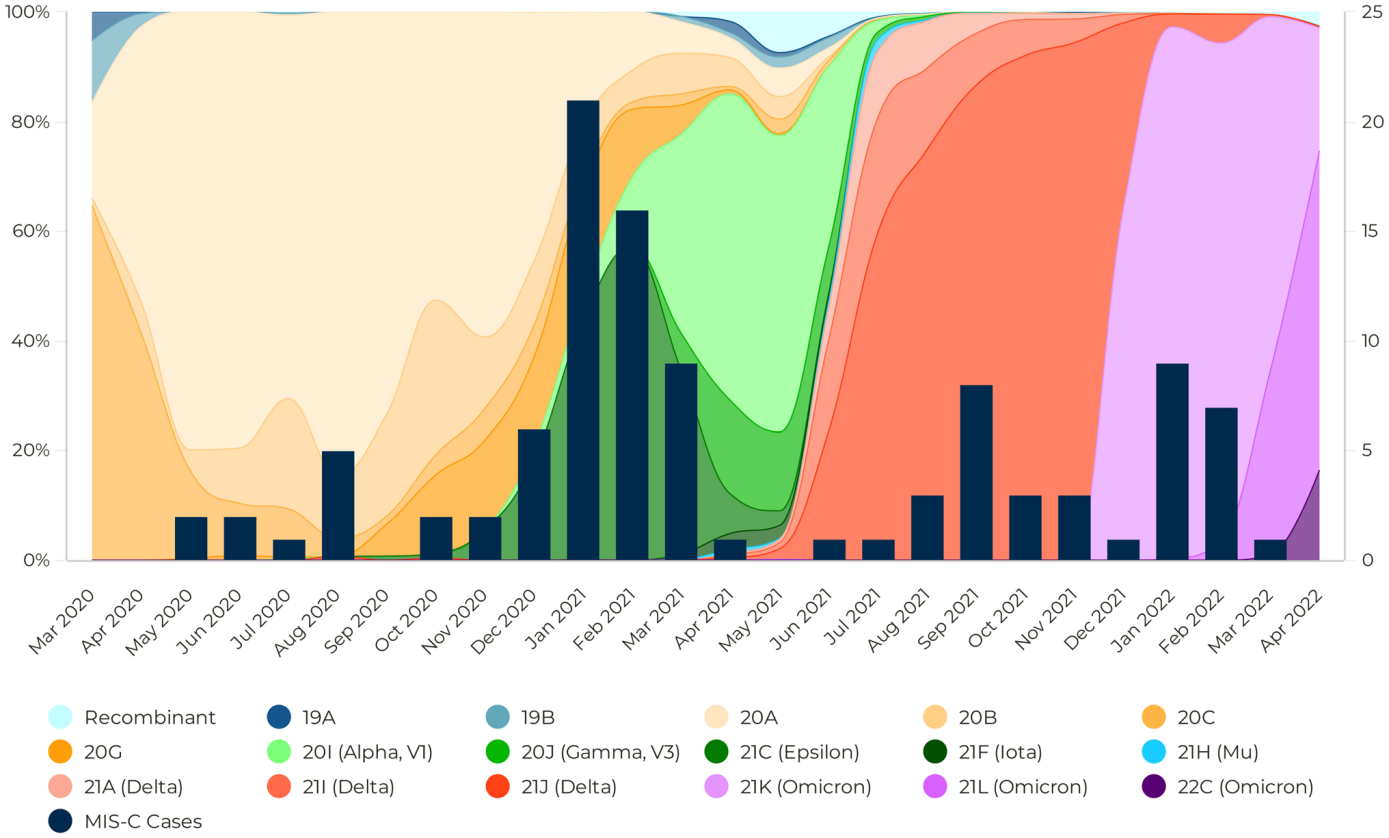
Circulating SARS‑CoV‑2 variants in San Diego (stacked area) and frequency of MIS-C cases at RCHSD (dark blue columns)

**Fig. 2:**
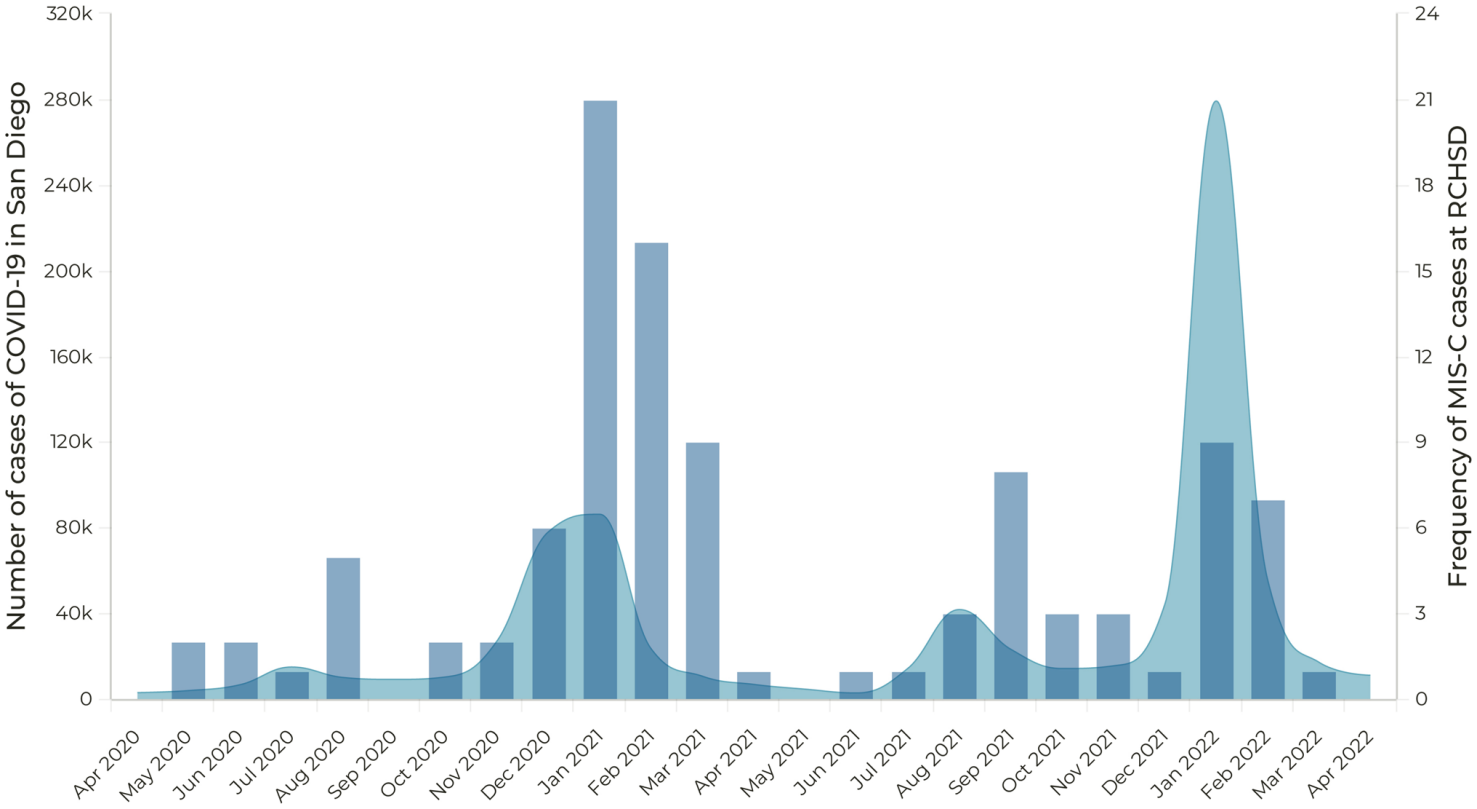
Number of cases of COVID in San Diego (area in light blue) and frequency of MIS-C cases at RCHSD (dark blue columns)

The data from https://github.com/andersen-lab/HCo-V-19-Genomics [19, 20] included 79,421 SARS‑CoV‑2 sequences from San Diego County obtained between March 2020 and November 2022. The Nextclade CLI could not align and analyze 559 sequences, which were excluded. Figure 1 shows the proportion of circulating SARS-CoV-2 variants in San Diego County over time with the number of MIS-C cases at RCHSD. Circulating viruses during the first MIS-C wave included clades 20A, 20B, and 20C. The second wave began with the same viruses but also when clade 20G started circulating, its peak coincided with the circulation of 21C (Epsilon) and faded with the appearance of clades 20I (Alpha, V1) and 20J (Gamma, V3). The third and fourth waves coincided with a predominant circulation of clades 21A, 21I and 21J (Delta strains) and clades 21K, 21L and 22C (Omicron variants), respectively. MIS-C usually presents 2–6 weeks after COVID infection, and as expected, the peak of waves of MIS-C occurred after the peak of the waves of COVID cases reported in San Diego (Fig. 2). Clades 20D, 20E (EU1), 20H (Beta, V2), 21B (Kappa), 21D (Eta), and 21G (Lambda) had low circulation (frequency less than 2% per month) during the period of study.

### Analysis of MIS-C waves

The demographics, presenting clinical signs, and laboratory values prior to treatment did not differ among the different waves (Table 1). None of the patients had hypertension, diabetes mellitus, congenital heart disease, chronic kidney disease, or autoimmune conditions. Only 6/104 (6%) patients had a history of asthma. The worst values during hospitalization for troponin I, BNP, ferritin, platelet count, or CRP were also not significantly different across the waves (Table 2).

**Table 1:** Demographic and clinical characteristics of MIS-C patients across the waves

Characteristics	Wave 1	Wave 2	Wave 3	Wave 4	All MIS-C cases	*P* value
*Number*	10	57	20	17	104	
Age (y)	10.1 (6.46–11.11)	8.23 (5.09–11.32)	7.76 (5.51–9.22)	6.31 (4.54–11.31)	7.95 (5.15–11.3)	0.71
Female	1 (10%)	26 (46%)	5 (25%)	4 (24%)	36 (35%)	0.07
Race/ethnicity						0.9
African American	2 (20%)	5 (9%)	3 (15%)	1 (6%)	11 (11%)	
Hispanic	6 (60%)	39 (68%)	11 (55%)	10 (59%)	66 (64%)	
Caucasian	1 (10%)	4 (7%)	1 (5%)	2 (12%)	8 (8%)	
More than one race	1 (10%)	7 (12%)	4 (20%)	3 (18%)	15 (14%)	
Other	0 (0%)	1 (2%)	0 (0%)	1 (6%)	2 (2%)	
Asian	0 (0%)	1 (2%)	1 (5%)	0 (0%)	2 (2%)	
Weight (kg)	38.45 (23.65–53)	37.3 (21.1–57)	32.55 (21.73–39.63)	25.7 (18–42.2)	32.55 (21.08–49.97)	0.42
Height (cm)	138.5 (118.1–153.33)	130 (111–153.5)	125.5 (118.5–138.75)	122 (108–143)	129.25 (113.5–149.25)	0.66
BMI	18.93 (16.14–24.47)	19.06 (16.57–25.67)	18.6 (15.75–20.79)	17.27 (16.06–19.51)	18.59 (16.14–24.05)	0.27
Percentile BMI	82.9 (54.9–96.3)	92.9 (59.08–97.65)	76.9 (58.68–87.55)	69.2 (32.35–94.15)	82.95 (51.3–97.18)	0.24
Overweight/obese	4 (40%)	31 (54%)	6 (30%)	5 (29%)	46 (44%)	0.14
Obesity	3 (30%)	22 (39%)	4 (20%)	3 (18%)	32 (31%)	0.28
Severe obesity	2 (20%)	8 (14%)	4 (20%)	0 (0%)	14 (14%)	0.2
Length of Stay	5 (4–6)	6 (4–8)	4 (4–6)	5 (4–7)	5 (4–7)	0.39
Clinical presentations						
Fever	10 (100%)	57 (100%)	20 (100%)	17 (100%)	104 (100%)	-
Rash	8 (80%)	34 (60%)	11 (55%)	12 (71%)	65 (63%)	0.51
Conjunctival injection	8 (80%)	44 (77%)	18 (90%)	14 (82%)	84 (81%)	0.69
Erythema of the lips, oral mucosa, pharynx	6 (60%)	29 (51%)	8 (40%)	11 (65%)	54 (52%)	0.49
Cervical lymphadenopathy	2 (20%)	9 (16%)	5 (25%)	2 (12%)	18 (17%)	0.71
Erythema of the hands	1 (10%)	11 (19%)	4 (20%)	2 (12%)	18 (17%)	0.88
Abdominal pain	9 (90%)	39 (68%)	14 (70%)	12 (71%)	74 (71%)	0.61
Emesis	7 (70%)	46 (81%)	14 (70%)	14 (82%)	81 (78%)	0.62
Diarrhea	7 (70%)	36 (63%)	9 (45%)	7 (41%)	59 (57%)	0.22

Continuous variables: Median (IQR). Categorical variables: *n* (%)

**Table 2:** Comparison of laboratory data across the four waves

Laboratory	Wave 1	Wave 2	Wave 3	Wave 4	All MIS-C cases	*P* value	Ref. values
Hematologic							
WBC (× 10^9^/L)	8.3 (4.95–11.2)	8.8 (6.6–13.2)	8.45 (6.88–10.7)	9.1 (6.1–12)	8.7 (6.5–12.08)	0.78	4.0–10.5
Hb (g/dL)	11 (10.53–12.08)	11.6 (10.5–12.2)	11.8 (10.6–12.35)	11.5 (10.7–12.3)	11.6 (10.58–12.23)	0.82	12.5–15.0
Platelet count (× 10^9^/L)	128 (91.75–226)	145 (98–206)	121.5 (100.75–151.5)	132 (107–160)	136 (97.75–182.25)	0.71	140–440
D dimer (mcg/mL)	2.34 (1.71–3.02)	3.49 (2.07–5.5)	3.27 (1.65–3.93)	2.2 (1.48–3.48)	3.27 (1.82–4.68)	0.16	< 0.5
Fibrinogen (mg/dL)	575 (502.5–676.75)	563 (469–632)	592 (483–638)	535 (491–719)	549 (483–641)	0.97	138–452
Inflammatory markers							
ESR (mm/hr)	41 (36.25–56.25)	35 (26.75–67.5)	33 (25–53)	26 (19.5–55.5)	35 (25.25–61.5)	0.31	0–20
CRP (mg/dL)	21 (19.65–26.05)	21.4 (15.4–28)	20 (14.18–24.83)	23.8 (19.6–29.9)	21 (16.58–27.92)	0.57	0–0.99
Ferritin (ng/mL)	603 (249.5–1004.25)	445.5 (259.25–885.75)	527 (388–1250)	360 (266.28–860.75)	469 (265–941)	0.67	6–70
Neutrophil to Lymphocyte ratio (NLR)	7.11 (3.59–12.21)	13.14 (6.46–23)	12.07 (7.55–17.55)	7.25 (3.67–15)	11.88 (6.1–18.45)	0.24	
Liver function							
ALT (U/L)	33 (22.25–56.75)	44 (27–63)	38 (29.25–60.5)	32 (25–44)	40 (26.75–61.25)	0.37	5–38
AST (U/L)	52 (42–79)	55 (39–77)	57 (42.5–82)	49 (36–67.75)	52 (41–77)	0.8	5–30
GGT (U/L)	43.5 (34.5–82)	36 (25–89)	40 (21–86)	24.5 (16–41.5)	36 (24–85.75)	0.17	11–28
Albumin (g/dL)	3.3 (2.88–3.7)	3.5 (3.18–3.8)	3.65 (3.35–3.95)	3.6 (3–4.2)	3.5 (3.1–3.9)	0.4	3.5–5.1
Renal function							
Sodium (mmol/L)	134 (131.5–135.75)	132 (129–134)	131.5 (129.75–133.25)	131 (128–134)	132 (129–134)	0.53	133–143
Creatinine (mg/dL)	0.5 (0.32–0.57)	0.46 (0.36–0.62)	0.45 (0.38–0.58)	0.41 (0.27–0.52)	0.45 (0.35–0.6)	0.47	0.6–1.2
Cardiac function							
BNP (pg/mL)	87 (30.75–320.25)	86 (19.5–311.5)	135 (17–544.25)	188 (45–620.25)	105 (20.25–342)	0.7	< 100
Troponin I (ng/mL)	0.02 (0.01–0.13)	0.01 (0.01–0.06)	0.02 (0.01–0.12)	0.01 (0.01–0.05)	0.01 (0.01–0.07)	0.91	< 0.05
Categorical values							
Leukopenia	3 (30%)	4 (7%)	1 (5%)	2 (11.8%)	10 (9.6%)	0.12	
Lymphopenia	8 (80%)	47 (82.5%)	19 (95%)	14 (82.4%)	88 (84.6%)	0.52	
Thrombocytopenia	6 (60%)	31 (54.4%)	14 (70%)	12 (70.6%)	63 (60.6%)	0.53	
Hypoalbuminemia	7 (70%)	26 (45.6%)	8 (40%)	8 (47.1%)	49 (47.1%)	0.5	
Hyponatremia	6 (60%)	43 (75.4%)	17 (85%)	13 (76.5%)	79 (76%)	0.5	
Elevated ferritin	7 (70%)	34 (59.7%)	14 (70%)	11 (64.7%)	66 (63.5%)	0.55	
Elevated NLR	7 (70%)	50 (87.7%)	18 (90%)	13 (76.5%)	88 (84.6%)	0.3	
Elevated troponin	3 (30%)	14 (24.6%)	7 (35%)	4 (23.5%)	28 (26.9%)	0.81	
Elevated BNP	0 (0%)	6 (10.5%)	2 (10%)	3 (17.7%)	11 (10.6%)	0.59	
Worst value during hospitalization							
Troponin I (ng/mL)	0.04 (0.01–0.18)	0.06 (0.02–0.43)	0.08 (0.03–0.23)	0.06 (0.01–0.13)	0.06 (0.02–0.29)	0.76	< 0.05
CRP (mg/dL)	21.95 (19.88–28.08)	25.1 (17.8–30.5)	21.95 (14.18–29.9)	24.5 (20.4–28.1)	24 (18.55–30.05)	0.41	0–0.99
Ferritin (ng/mL)	853 (403.75–1064.75)	602 (303–1116)	524.5 (376–1296)	609.5 (256.75–1277)	601 (310–1116)	0.96	6–70
Platelet count (× 10^9^/L)	103.5 (88.25–188)	117 (87–171)	114 (98.5–140.25)	120 (79–156)	116.5 (87.75–156)	0.99	140–440
BNP (pg/mL)	478.5 (82.75–739.5)	496 (227–1592)	300.5 (150–664)	605 (119–1407)	456.5 (191.25–1264.5)	0.46	< 100
Thrombocytopenia	7 (70%)	41 (71.9%)	16 (80%)	12 (70.6%)	76 (73.1%)	0.89	
Elevated ferritin	9 (90%)	43 (75.4%)	17 (85%)	10 (58.8%)	79 (76%)	0.64	
Elevated troponin	4 (40%)	29 (50.9%)	12 (60%)	9 (52.9%)	54 (51.9%)	0.79	
Elevated BNP	2 (20%)	21 (36.8%)	3 (15%)	7 (41.2%)	33 (31.7%)	0.21	

Continuous variables: median (IQR). Categorical variables: *n* (%)

*WBC* white blood cell count, *Hb* hemoglobin, *ESR* erythrocyte sedimentation rate, *CRP* C reactive protein, *ALT* alanine transaminase, *AST* aspartate aminotransferase, *GGT* gamma-glutamyl transferase, *BNP* brain natriuretic peptide

Overall, 79/104 (76%) presented with hyponatremia, 63/104 (61%) with thrombocytopenia, 49/103 (47%) with hypoalbuminemia, and 88/104 (85%) with lymphopenia on admission. Elevated troponin levels were not significantly different among the groups on admission or during hospitalization. For all the waves, 28/100 (28%) had elevated troponin on admission, but the number increased to 54/104 (52%) during hospitalization. Half of the patients (52/102, 51%) presented with an elevated BNP (≥ 100 pg/mL), which increased to 85/104 (82%) during the hospital course. A markedly elevated BNP value of ≥ 1000 pg/mL was noted in 11/102 (11%) of patients on admission and increased to 33/104 (32%) during the hospital stay, although the percentage of patients was not different across variant groups.

As a measure of clinical severity, the need for intensive care differed across the waves, with more patients admitted to the PICU and more patients requiring inotropic support during the second wave (Table 3). The median length of stay in the PICU and the need for respiratory support were not significantly different among the waves. Only two patients required mechanical ventilation, and none required extracorporeal circulatory support. The median volume of IV fluid boluses administered during the first 24 hours of admission was about 20 mL/kg for all waves. There were also no significant differences in the use of diuretics. All waves had a similar proportion of patients with cardiac dysfunction at the time of admission. Throughout hospitalization, the second wave had more patients with cardiac dysfunction (61%) than the other waves (35% to 47%). However, no statistical significance was reached (*P* = 0.19). The median *Z* score for the worst coronary artery measurement was similar among all waves. Most patients presented with either normal coronary artery dimensions (*Z* score < 2.0, 71%) or mild coronary artery dilation (*Z* score 2.0–3.0, 18%). There were no patients with large or giant coronary aneurysms. The median time from admission to initiation of the IVIG infusion was 9.95 hours, with no significant differences across the wave groups. There were no differences in the number of anti-inflammatory therapies received (Table 3).

**Table 3:** Comparison of clinical support, echocardiographic findings, and management across the four waves

Variables	Wave 1	Wave 2	Wave 3	Wave 4	All MIS-C cases	*p* value
PICU admission	3 (30%)	42 (73.7%)	9 (45%)	9 (52.9%)	63 (60.6%)	0.01*
Days in the PICU	4 (4–5.5)	3.5 (2–5)	2 (2–4)	3 (2–5)	3 (2–5)	0.3
Inotropic support	2 (20%)	36 (63.2%)	7 (35%)	7 (41.2%)	52 (50%)	0.02*
Norepinephrine	1 (10%)	6 (10.5%)	1 (5%)	2 (11.8%)	10 (9.6%)	0.91
Epinephrine	1 (10%)	31 (54.4%)	7 (35%)	7 (41.2%)	46 (44.2%)	0.05*
Milrinone	1 (10%)	29 (50.9%)	4 (20%)	6 (35.3%)	40 (38.5%)	0.02*
Respiratory support						
None	7 (70%)	44 (77.2%)	17 (85%)	14 (82.4%)	82 (78.8%)	0.75
Nasal cannula	1 (10%)	5 (8.8%)	1 (5%)	0 (0%)	7 (6.7%)	
NIPP	2 (20%)	7 (12.3%)	1 (5%)	3 (17.7%)	13 (12.5%)	
Intubated	0 (0%)	1 (1.8%)	1 (5%)	0 (0%)	2 (1.9%)	
*LV systolic function*^*a*^						
LVEF < 55% on admission	2 (20%)	19 (33.3%)	6 (30%)	6 (35.3%)	33 (31.7%)	0.96
LVEF < 55% during hospitalization	4 (40%)	35 (61.4%)	7 (35%)	8 (47.1%)	54 (51.9%)	0.19
LVEF < 50% during hospitalization	3 (30%)	24 (42.1%)	5 (25%)	7 (41.2%)	39 (37.5%)	0.58
Worst LVEF (%)	56 (49–60)	53 (44–60)	58.5 (49–61)	55 (38–57)	53 (46.5–60)	0.6
*Coronary arteries*^*a*^						
Z-score < 2	4 (40%)	44 (77.2%)	14 (70%)	12 (70.6%)	74 (71.2%)	0.26
Z-score 2 to < 3	3 (30%)	9 (15.8%)	5 (25%)	2 (11.8%)	19 (18.3%)	
Z-score > = 3	2 (20%)	4 (7%)	1 (5%)	3 (17.7%)	10 (9.6%)	
Worst Coronary Z score	2.07 (1.44–2.52)	1.44 (1.04–1.93)	1.44 (0.88–2.04)	1.6 (1.4–2.47)	1.56 (1.08–2.15)	0.27
Fluid boluses first 24 h of admission						
ml/kg	20 (12.5–30)	20 (10–30)	21.5 (15.25–40)	20 (10–30)	20 (10–30.38)	0.95
ml/BSA	681.32 (386.6–1030.8)	632.86 (395.9–866.9)	688.83 (406.1–1063.2)	495.63 (279.9–846.4)	623.94 (371.4–1027.4)	0.85
Diuretics	3 (30%)	28 (49.1%)	5 (25%)	5 (29.4%)	41 (39.4%)	0.18
IVIG	9 (90%)	56 (98.3%)	19 (95%)	17 (100%)	101 (97.1%)	0.32
Hours from admission to order	10.5 (3.88–18.95)	6.55 (2.92–10.64)	12.58 (5.82–17.09)	7.12 (4.85–10.68)	7.05 (4.05–13.35)	0.20
Hours from admission to administration	12.98 (5.92–20.42)	8.44 (6.51–14.36)	13.87 (7.84–21.6)	10.43 (6.57–13.3)	9.95 (6.63–16.38)	0.32
Other anti-inflammatory therapies						
Steroid	7 (70%)	36 (63.2%)	10 (50%)	11 (64.7%)	64 (61.5%)	0.7
Infliximab	4 (40%)	29 (50.9%)	13 (65%)	7 (41.2%)	53 (51%)	0.44
Anakinra	1 (10%)	31 (54.4%)	7 (35%)	8 (47.1%)	47 (45.2%)	0.05
Number of anti-inflammatory medications^b^	2 (2–2.75)	3 (2–3)	2 (2–3)	2 (2–3)	3 (2–3)	0.31

Continuous variables: Median (IQR). Categorical variables: *n* (%). *PICU* Pediatric intensive care unit, *NIPP* Non-invasive positive pressure ventilation (includes high flow nasal cannula, Continuous positive airway pressure, and Bilevel positive airway pressure). *LV* Left ventricle, *LVEF* Left ventricular ejection fraction, *BSA* Body surface area

^a^One patient had no echo during the admission on wave 1

^b^The number of anti-inflammatory therapies included steroids, IVIG, Infliximab, and Anakinra

*Pairwise comparisons failed to demonstrate statistically significant differences between specific groups, likely related to the size of the sample

## Discussion

SARS-CoV-2 has mutated throughout the pandemic, changing its infectivity and clinical presentation. Little is known about how these mutations affect delayed COVID complications such as MIS-C. We divided the waves based on the local rate of MIS-C cases and utilized local SARS-CoV-2 sequence data to compare MIS-C cases with contemporaneous circulating variants. The San Diego experience showed that patients affected with MIS-C presented initially with similar symptoms and laboratory findings across all the variant waves. However, the severity of MIS-C differed, with more severe cases presenting when a mixture of variants (clades 20A-C, 20G, 21C Epsilon, 20I Alpha, and 20J Gamma) was circulating. These patients required more admission to the PICU and inotropic support. Different from what has been reported in other studies, the second wave was a combination of several variants.

Raju Abraham et al. reported their experiences in Cape Town, South Africa, with similar wave dates to our cohort [9]. They found no significant differences across groups in the clinical presentation, laboratory features, or disease course. However, the management was heterogeneous. Levy et al. described the experience in Israel during the most recent three waves [11]. They performed a prospective study in 12 Israeli hospitals. The group admitted during the Omicron wave had a shorter length of stay, required fewer vasopressors, had lower NT-proBNP levels, reduced need for mechanical ventilation, and fewer patients with an LVEF of less than 40%. There was also a lower MIS-C incidence rate during the Omicron wave. Ganguly et al. published their experience with the “first and second waves” in Eastern India, with the Delta variant causing a more severe presentation with poorer outcomes [10]. The management between those waves differed, with fewer patients receiving IVIG therapy during the Delta wave and more receiving steroids. The reported experience in all hospitals in Catalonia, Spain, by Pino et al., did not demonstrate differences in phenotype and severity throughout the pandemic [13]. At the University Children’s Hospital of Cracow in Poland, Ptak et al. did not demonstrate changes in the clinical course of MIS-C [14]. However, the authors analyzed the patients with only the “Original/Alpha” and “Delta/Omicron” variants groups.

In the United States, Harahsheh et al. also described their experience with the first two waves in Washington, DC, with a more standardized treatment [7]. For patients in wave 2, they found a higher proportion of children older than 15 years, higher median troponin I and BNP values, greater need for vasopressors and anti-inflammatory therapies, and a higher rate of admission to the PICU. Despite these differences, systolic function, coronary *Z* scores, and length of stay were similar. All patients were discharged home at a median of 11 days. Jain et al. reported the experience in Houston, Texas [8]. They merged the first two waves (original and alpha variant cohorts) and compared them against the third wave (delta variant cohort). In the original/alpha cohort, there were more males, more respiratory and musculoskeletal symptoms on presentation, longer PICU stays, and a greater need for mechanical ventilation/ECMO/LV assist devices. The groups also differed in median values for INR, PT, WBC, sodium, phosphorus, and potassium. Miller et al. analyzed data from local, state, and territorial health departments reporting cases across the United States [5, 6]. The classification of the waves was based on the trends in MIS-C cases but not on the circulating variants. They noted a trend toward decreased MIS-C severity over time, including less cardiac dysfunction, shorter hospital and PICU stays, and decreased case fatality. Last, Laird-Gion et al. published data from Boston, Massachusetts, sorting the patients into three cohorts (“Alpha”, “Delta”, and “Omicron”) based on national and regional data of variant prevalence [12]. More patients had a documented history of COVID-19 infection in the two months before presenting with MIS-C in the “Omicron” cohort than in the “Alpha” cohort. Except for the lowest counts of platelets and absolute neutrophils during “Omicron”, there were no differences in other laboratories and markers of clinical severity (ICU admission, length of stay, use of inotropes, LV dysfunction) across the different variants.

SARS‑CoV‑2 spread worldwide, and acute infection occurred at different times in different locations. Some variants were more prevalent only in certain regions. These factors might explain part of the differences noted in the reported studies. However, the definition and analysis of waves, groups, and cohorts have not been standardized among those studies (Table 4).

**Table 4:**
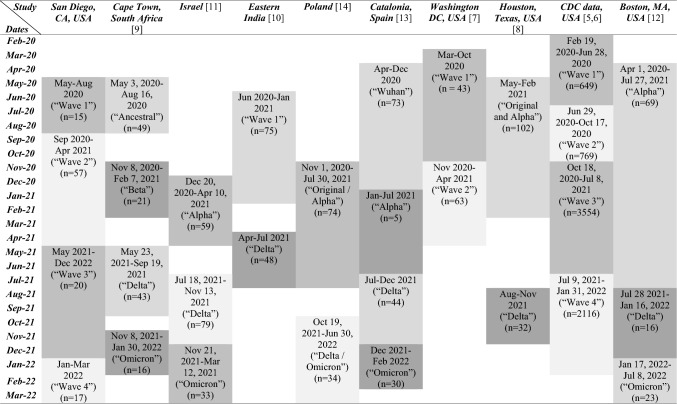
Comparison of periods used in studies evaluating differences across MIS-C waves

Compared with the experience in other centers, none of our patients required ECMO or ventricular assist devices, and only two needed mechanical ventilation. One required intubation for a sedated procedure; the other was transferred from another institution after receiving extensive volume resuscitation and presented with pulmonary edema and cardiogenic shock. Our approach was to initiate IVIG therapy as quickly as possible and avoid excessive fluid resuscitation in the first 24 h. We also report a shorter hospital length of stay than other single-center studies and more aligned with the US national surveillance data. After the wave related to Omicron clades 21K and L, we saw only rare sporadic cases of MIS-C. This observation suggests that as the virus becomes endemic and variants cause milder symptoms with initial infection, coupled with the increasing vaccination rate among children and large percentage of previously infected children, perhaps MIS-C will become less prevalent.

### Strengths and limitations

A single team with extensive experience in Kawasaki disease guided the management of MIS-C patients, allowing rapid diagnosis and standardized management of children early in the pandemic. The analysis was based on sequencing of variants in the same community as the patients, which allowed a detailed analysis of the clinical impact of each variant wave. All patients had serologic evidence of previous COVID-19 infection. However, we could not attribute specific SARS-CoV-2 genomic information to each patient. As the samples were not obtained randomly, there is a possibility of bias. This is a single-center study, with data collected from the electronic medical record and all the limitations associated with a retrospective study. The low number of patients in some of the waves limited the power of the statistical analysis.

In conclusion, this study suggests that various strains of SARS-CoV-2 trigger MIS-C with differing severities. The second wave had several circulating clades in San Diego: 20A to C, 20G, 21C (Epsilon), 20I (Alpha), and 20J (Gamma). Those patients had a more severe clinical course than patients in other waves. Overall, patients in this series did well. Judicious fluid management and prompt initiation of IVIG therapy might have contributed to the shorter length of stay and less need for mechanical cardiovascular and invasive respiratory support reported in this study. Whether the differences in disease severity across variants were due to changes in the virus or other factors, including previous infection, remains unknown.

## Data Availability

The COVID-19 epidemiologic data for San Diego County is publicly available from https://searchcovid.info/dashboards/epidemiology/ and https://outbreak.info. The local genomic data is also publicly available at https://github.com/andersen-lab/HCo-V-19-Genomics. These databases are updated regularly. The datasets containing specific patient information are in a secured data storage system (Research Electronic Data Capture, REDCap) managed by the Kawasaki Disease Research Center, La Jolla, CA, USA. They are not openly available as they contain protected health information. De-identified records might be available from the corresponding author upon reasonable request.
